# Implementation of the Goal-directed Medication review Electronic Decision Support System (G-MEDSS)© into home medicines review: a protocol for a cluster-randomised clinical trial in older adults

**DOI:** 10.1186/s12877-020-1442-2

**Published:** 2020-02-12

**Authors:** Lisa Kouladjian O’Donnell, Mouna Sawan, Emily Reeve, Danijela Gnjidic, Timothy F. Chen, Patrick J. Kelly, J. Simon Bell, Sarah N. Hilmer

**Affiliations:** 1 NHMRC Cognitive Decline Partnership Centre, Northern Clinical School, Faculty of Medicine and Health, The University of Sydney, Sydney, NSW Australia; 2 Departments of Clinical Pharmacology and Aged Care, Kolling Institute of Medical Research, Royal North Shore Hospital, St Leonards, New South Wales 2065 Australia; 3 Geriatric Medicine Research, Faculty of Medicine, and College of Pharmacy, Dalhousie University and Nova Scotia Health Authority, Halifax, Canada; 4 College of Medicine, University of Saskatchewan, Saskatchewan, Canada; 5 Quality Use of Medicines and Pharmacy Research Centre, School of Pharmacy and Medical Sciences, Division of Health Sciences, University of South Australia, Adelaide, South Australia Australia; 6 Sydney Pharmacy School, Faculty of Medicine and Health, The University of Sydney, Sydney, NSW Australia; 7 Charles Perkins Centre, The University of Sydney, Sydney, NSW Australia; 8 School of Public Health, Faculty of Medicine and Health, The University of Sydney, Sydney, NSW Australia; 9 Centre for Medicine Use and Safety, Faculty of Pharmacy and Pharmaceutical Sciences, Monash University, Parkville, Victoria Australia; 10 School of Public Health and Preventive Medicine, Faculty of Medicine, Nursing and Health Sciences, Monash University, Melbourne, Victoria Australia; 11 Sansom Institute, School of Pharmacy and Medical Sciences, University of South Australia, Adelaide, South Australia Australia

**Keywords:** Deprescribing, Drug burden index, Dementia, Older adults, Patient centred care, Home medicines review

## Abstract

**Background:**

Older people living in the community have a high prevalence of polypharmacy and are vulnerable to adverse drug events. Home Medicines Review (HMR) is a collaborative medication review service involving general practitioners (GPs), accredited clinical pharmacists (ACPs) and patients, which aims to prevent medication-related problems. This study aims to evaluate the implementation of a Computerised Clinical Decision Support System (CCDSS) called G-MEDSS© (Goal-directed Medication Review Electronic Decision Support System) in HMRs to deprescribe anticholinergic and sedative medications, and to assess the effect of deprescribing on clinical outcomes.

**Methods:**

This study consists of 2 stages: Stage I – a two-arm parallel-group cluster-randomised clinical trial, and Stage II – process evaluation of the CCDSS intervention in HMR. Community-dwelling older adults living with and without dementia who are referred for HMR by their GP and recruited by ACPs will be included in this study. G-MEDSS is a CCDSS designed to provide clinical decision support for healthcare practitioners when completing a medication review, to tailor care to meet the patients’ goals and preferences. The G-MEDSS contains three tools: The Goals of Care Management Tool, The Drug Burden Index (DBI) Calculator©, and The revised Patients’ Attitudes Towards Deprescribing (rPATD) questionnaire. The G-MEDSS produces patient-specific deprescribing reports, to be included as part of the ACPs communication with the patient’s GP, and patient-specific reports for the patient (or carer). ACPs randomised to the intervention arm of the study will use G-MEDSS to create deprescribing reports for the referring GP and for their patient (or carer) when submitting the HMR report. ACPs in the comparison arm will provide the usual care HMR service (without the G-MEDSS).

**Outcomes:**

The primary outcome is reduction in DBI exposure 3 months after HMR ± G-MEDSS intervention between comparison and intervention groups. The secondary outcomes include changes in clinical outcomes (physical and cognitive function, falls, institutionalisation, GP visits, medication adherence and mortality) 3-months after HMR.

**Discussion:**

This study is expected to add to the evidence that the combination of CCDSS supporting medication review can improve prescribing and clinical outcomes in older adults.

**Trial registration:**

The trial was registered on the Australian New Zealand Clinical Trials Registry ACTRN12617000895381 on 19th June 2017.

## Background

Polypharmacy is increasingly common in adults aged 65 years and over, internationally. In a large-scale cross-sectional analysis of Scottish prescribing data, the proportion of individuals prescribed five or more regular medications has increased from 11.4 to 20.8% between 1995 and 2010 [1]. In Australia, polypharmacy is identified in approximately 37.7–43.3% of older adults living in the community, and has been associated with adverse drug events (ADEs) including falls, hospitalisation, mortality, and declining physical and cognitive function [2–4]. Inappropriate prescribing, commonly defined as when medications introduce a significant risk of an ADE when there is evidence for an equally more effective treatment, may also contribute to polypharmacy [5]. Deprescribing, which is the process of withdrawal of an inappropriately prescribed medication with medical supervision, has the potential to reduce polypharmacy and improve outcomes in older adults [6].

Optimising medication management in older people living with dementia is particularly complex, as dementia is commonly associated with multimorbidity, and as a consequence polypharmacy and ADEs [7, 8]. Worldwide, approximately 50 million people are living with dementia [9]. Among Australians aged 65 years and over, 10% have dementia, and the prevalence of dementia increases to 31% of Australians aged 85 years and over [10]. Studies have shown that community-dwelling people living with dementia are prescribed more medications than people without dementia, and may be more vulnerable to ADEs [11, 12]. For instance, people living with dementia are especially sensitive to ADEs associated with CNS-acting medications, and there is an increased risk of mortality in people with Alzheimer’s disease who are prescribed an antipsychotic medication [13, 14].

**Fig. 1:**
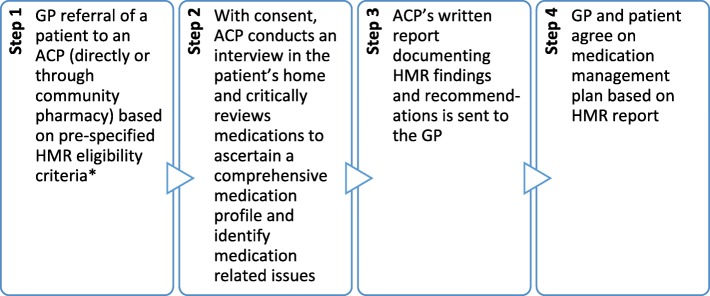
The process of a Home Medicines Review (HMR) in Australia. ACP = Accredited Clinical Pharmacist; GP = General Practitioner. *For a patient to be eligible for a HMR they must: be a current Australian Medicare/Department of Veterans’ Affairs cardholder, live in a community setting, and be at risk of experiencing medication misadventure as determined by their prescribing doctor [16].

The Home Medicine Review (HMR) service is an Australian government-funded pharmacist-led medication review service for patients living in the community setting. The service aims to reduce medication-related problems, medication-related hospital admissions, and improve the responsible use of medicines for patients [15]. The HMR model is a collaborative service between the patient, general practitioner (GP) and accredited clinical pharmacist (ACP). An ACP is a specially trained and credentialed clinical pharmacist who has received post-registration certification in medication review. The HMR involves the identification and documentation of actual and potential causes of medication-related problems by the ACP, and presenting recommendations to resolve these in a written report to the GP to inform the patient’s medication management plan (Fig. 1) [16, 17]. The HMR presents an opportunity to plan and commence deprescribing of inappropriate medications in older adults. International studies have demonstrated that medication reviews improve patient medication knowledge and adherence, and appropriateness of prescribed medications [18, 19]. In people living with dementia, pharmacist-led medication management services have been shown to improve quality use of medicines, quality of life and health outcomes [20]. Recent systematic reviews have concluded that although pharmacist-led medication review may be beneficial in improving medication-related problems, effects on patient health outcomes such as quality of life, hospitalisation and mortality is less clear [19].

Computerised Clinical Decision Support Systems (CCDSS) have the potential to improve GPs’ and pharmacists’ decision-making during the medication review process [21]. CCDSS apply algorithms to individual patient data to improve clinical decision making and optimise health outcomes, and may be considered as an enabler to deprescribing [22, 23]. For example, an interventional study of patients in a geriatric ward in Italy evaluated the effectiveness of a CCDSS in reducing potentially inappropriate medications. The use of a CCDSS in this study was associated with a significant reduction in potentially inappropriate medications and new onset of severe drug-drug interactions [24]. In a recent study, integration of a CCDSS into HMR was demonstrated as a feasible and useful method to prompt deprescribing of anticholinergic and sedative medications in older adults [25]. Although the implementation of CCDSS interventions into practice have improved medication prescribing, the magnitude of the effect is varied according to study design, and only a limited number of studies reported improved clinical outcomes [26, 27]. Furthermore, CCDSS platforms allow the incorporation of various validated tools to support practitioners when deprescribing. These can include tools to identify inappropriate medications, enhance shared decision-making and support goal-directed care.

The Drug Burden Index (DBI) was developed to pharmacologically measure the cumulative exposure of older adults to anticholinergic and sedative medications and relate this exposure to physical and cognitive function [28]. Increasing DBI scores have been associated with poorer physical function, falls, frailty, hospitalisation and mortality in older adults [29, 30]. The DBI Calculator© was developed as a reliable and valid CCDSS to report DBI of older patients taking multiple medications [31]. The DBI Calculator was recently investigated as a CCDSS in the HMR setting and demonstrated that it was a feasible and useful method to prompt deprescribing in older adults [25].

Clinicians have increasingly been encouraged to involve patients in the clinical decision-making process. In 2001, The United States Institute of Medicine defined patient-centred care as ‘care that is respectful of and responsive to individual patient preferences, needs and values’. [32] Recent studies on deprescribing have stressed the importance of identifying patients’ preferences, patient involvement and shared-decision making [33]. The revised Patients’ Attitudes Towards Deprescribing (rPATD) questionnaire explores peoples’ attitudes, beliefs, and experiences regarding the number of medications that they are taking and how they would feel about ceasing one or more of their medications [34]. This questionnaire identifies the barriers and enablers to deprescribing inappropriate medications at the individual patient level, is reliable, and has been validated in older patients, caregivers and people living with mild cognitive impairment and mild-to-moderate dementia (rPATDCog) [35]. The development of the rPATDCog demonstrated a strong agreement between responses from people living with cognitive impairment and their carers [35]. In recent studies, 60–80% of older adults and caregivers were willing to have a medication deprescribed if their doctor said it was possible [36, 37]. To date, the rPATD has not been trialled as a tool to guide deprescribing in a clinical setting.

Discussing the goals of care with older adults may help healthcare practitioners incorporate the concerns and wishes of patients when making decisions [38]. Goals of care are particularly important for people living with dementia, as dementia and its related comorbidities may be multifactorial, and goals of care may be less clear and less well articulated than for people without dementia [39]. Identifying and discussing goals of care during a HMR has the potential to guide prescribers in choosing appropriate treatment or care options for the individual.

We hypothesise that the combination of pharmacist-led medication review (HMR) and a CCDSS intervention that incorporates validated deprescribing tools and patient-centred guides may reduce the proportion of older adults using anticholinergic and sedative medications and improve clinical outcomes in community-dwelling older adults.

### Aims

Overall, this study aims to evaluate the implementation of a CCDSS in HMRs to deprescribe medications, particularly those with anticholinergic and sedative effects, and to assess the effect of deprescribing on prescribing and clinical outcomes. Specifically, this study will aim to:

- Reduce the proportion of patients who are exposed to anticholinergic and sedative medications as measured by the DBI;
- Examine the effect on clinical outcomes (including cognitive and physical function, falls, and institutionalisation), and mortality;
- Examine the effect on patient process outcomes (including adherence and physician visits)
- Evaluate the process of implementing a CCDSS intervention within HMR.

## Methods

### Study design

**Fig. 2:**
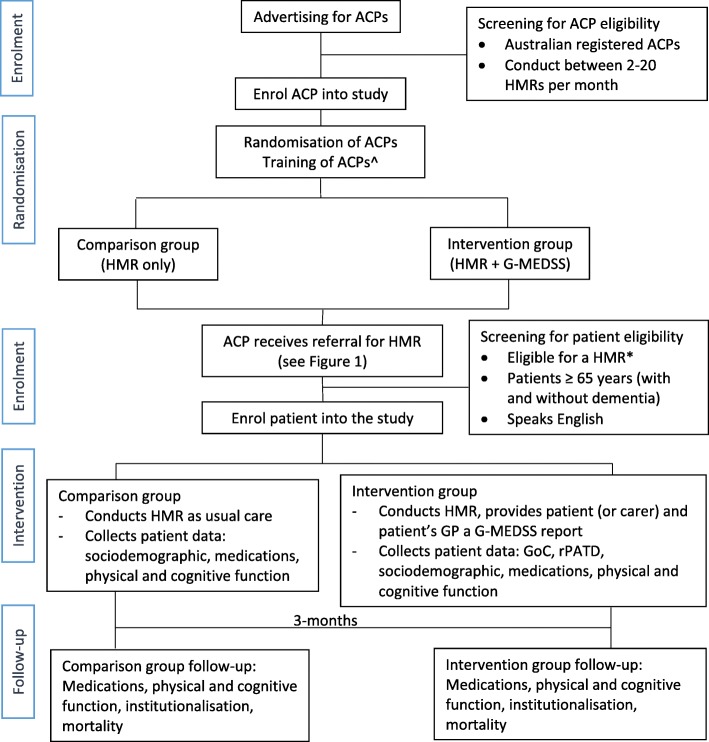
Study design. ACP = Accredited Clinical Pharmacist; HMR = Home Medicines Review; G-MEDSS = Goal-directed Medication review Electronic Decision Support System; GP = General Practitioner; GoC = Goals of Care; rPATD = revised Patients Attitudes Towards Deprescribing. ^Training will include: how to invite and collect patient/carer consent, how to collect data using the data collection sheets, and G-MEDSS training (for intervention group only). *For a patient to be eligible for a HMR they must: be a current Australian Medicare/Department of Veterans’ Affairs cardholder, live in a community setting, and be at risk of experiencing medication misadventure as determined by their prescribing doctor [16].

This study will be performed in two stages. Stage I consists of the cluster-randomised clinical trial, and Stage II will be the process evaluation of the implementation of the CCDSS in the HMR service. Stage I will be conducted as a two-arm, parallel group, cluster-randomised clinical trial, with the cluster assignment occurring at the level of the ACP (Fig. 2). Stage II will use quantitative and qualitative research (mixed-methods process evaluation) to evaluate the process of the intervention within HMR. Stage II will run alongside (in parallel to) Stage I.

#### Stage I: cluster-randomised clinical trial

##### Setting

The HMR is a community-based service and may be conducted within the urban or rural setting. ACPs (cluster unit) who conduct HMRs in Australia will be invited to participate in the study.

##### Recruitment of pharmacists

Australian registered ACPs who have conducted at least 24 HMRs in the last 12 months, and conduct 2–20 HMRs per month, will be eligible to participate in this study. We will advertise for ACPs through e-newsletters of professional organisations (e.g. Australian Association of Consultant Pharmacy, Society of Hospital Pharmacy, Pharmaceutical Society of Australia, the Australian Deprescribing Network, Sydney North Primary Health Network, and the Australian Journal of Pharmacy) and by providing expression of interest leaflets to delegates at the annual conferences of these professional organisations.

##### Recruitment of older people

ACPs who are enrolled in the study and have completed the training to participate in the study (see ‘*Study procedure: training’)* will then screen and recruit people living with and without dementia, who are sequentially referred to them for a HMR. Eligibility for individual people living with and without a diagnosis of dementia include: aged 65 years and older who can speak English, are eligible for a HMR, and are able to provide informed written consent (patient or carer).

##### Consent

**Fig. 3:**
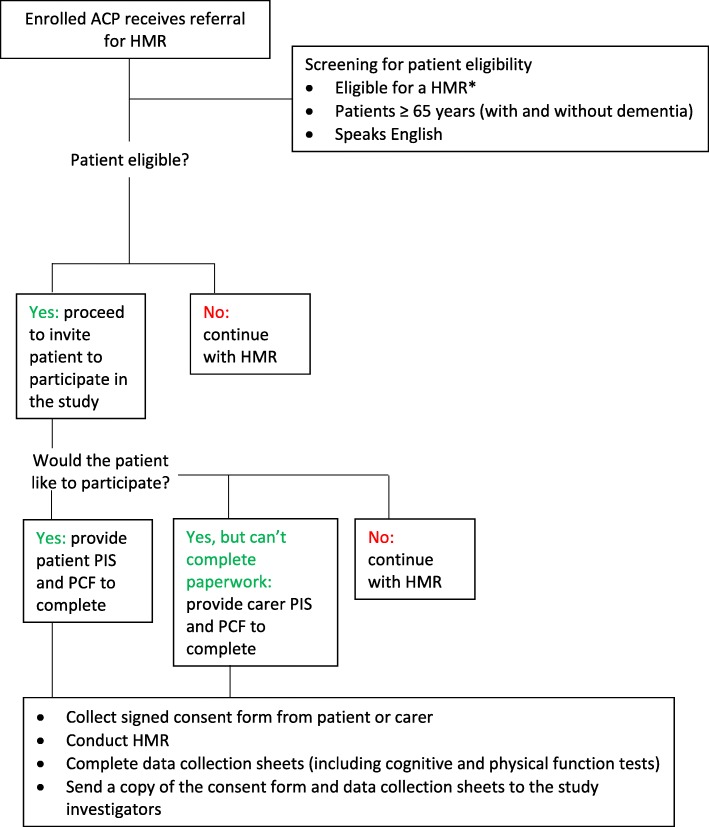
Process flow for pharmacists in study^. ACP = Accredited Clinical Pharmacist; HMR = Home Medicines Review; PIS = Participant Information Sheet; PCF = Participant Consent Form. ^irrespective of whether pharmacist is randomised into intervention (i.e. with G-MEDSS) or comparison group. *For a patient to be eligible for a HMR they must: be a current Australian Medicare/Department of Veterans’ Affairs cardholder, live in a community setting, and be at risk of experiencing medication misadventure as determined by their prescribing doctor [16].

All study participants will provide informed written consent. If a person who is referred to an ACP for a HMR meets the study’s eligibility requirements, a standardised verbal invitation will be extended by the ACP to the patient to participate in the study. For people living with cognitive impairment or mild-to-moderate dementia, consent from the patient will be obtained from the patients if they have the capacity to consent: the ACP will explain the study in simple language and ask to repeat their involvement in the study back to the ACP. Given their specialised training, and based on the patient’s responses, ACPs will be able to recognise whether the person has impaired cognition, and whether the patient’s carer should be approached for consent. If the person (or carer on behalf of the person) wishes to participate, the ACP will then obtain written consent from the person (or carer on behalf of the person). ACPs will be trained to follow the principles as determined by the Australian National Statement for Ethical Conduct in Human Research [40]. The HMR service will continue whether or not the person chooses to participate in the study (Fig. 3).

##### Randomisation and blinding

ACPs will be randomised to the intervention or comparison groups after the ACPs volunteer, eligibility is checked, and consent is given to participate in the study. An online random number generator will be used to formulate a data sheet of ACP allocations to either the intervention or comparison groups [41]. ACPs and patients will not be blinded to the study group, however ACPs will be advised to not communicate with other enrolled ACPs about the study.

#### Study procedure

##### Training

ACPs who have consented to be involved in the study will undergo a mandatory 2-h self-directed training program. This will involve training in the following components:

- Screening potential HMR recipients and obtaining written consent from people living with and without dementia for participation in this study;
- Using the data collection sheets to collect data from patients;
- Identifying and reporting adverse events (if required);
- Assessing physical performance of the patient using the Short Physical Performance Battery (SPPB) (training video available to download from https://www.irp.nia.nih.gov/branches/leps/sppb/).

ACPs randomised into the intervention arm will also receive training on how to use and implement the CCDSS into HMR. This training program has been designed by the investigators and based on a previous study [25]. All ACPs will be tested for their competency to participate in the trial by completing 10–15 multiple-choice questions (MCQs), with a pass mark of 70%. ACPs who do not meet the pass mark will be allowed to complete the competency MCQs again until they achieve the pass mark.

##### Intervention

The Goal-directed Medication review Electronic Decision Support System (G-MEDSS) (accessible from gmedss.com) is a validated electronic platform (CCDSS) that has been designed to provide clinical decision support for healthcare practitioners (GPs and ACPs) when completing a medication review for their older patients, to provide person-centred care to meet their goals and preferences. The G-MEDSS platform was, developed, validated and evaluated in a previous study with ACPs, GPs and carers for people living with dementia, using mixed-methodology that informed the design and usability of the tools within the CCDSS [42]. The G-MEDSS is designed to produce patient-specific deprescribing reports for a patient’s GP.

The G-MEDSS contains three tools:

1. The Goals of Care Management tool allows the patient’s goals of care to be identified and aligned with their medication management strategies. The tool also allows for free-text information to be entered into the system.
2. The Drug Burden Index Calculator© is a validated tool that identifies medications with anticholinergic and sedative effects (measured by the DBI) and provides information on a patient’s total exposure to these drug classes and their risks to older people [28, 31].
3. The revised Patient’s Attitudes Towards Deprescribing questionnaire (rPATD) was developed and validated to explore people’s attitudes, beliefs, and experiences regarding the number of medications that they are taking, and how they would feel about ceasing one or more of their medications [34, 43]. Three different versions of the rPATD were incorporated into G-MEDSS to allow for selection as appropriate for the patient: a) older adult, b) caregiver, and c) people with cognitive impairment (rPATDcog) versions [35]. The psychometric properties of the rPATD were established in Australian older adults and carers, and has established face, content, criterion, construct and internal validity and test-retest reliability.

The three tools within G-MEDSS will assist users to incorporate the patient’s goals, perspectives on deprescribing medications and their DBI score, together with their recommendations from their medication review, allowing a patient-centred approach to medication management. The G-MEDSS deprescribing report includes a combination of the results from the three tools: the person’s global goal of care, goals and strategies to improving the medication regimen, a summary of the patient’s current medications, the patient’s DBI score and information about interpreting the DBI, information about the rPATD, and a summary of the patient’s responses to the rPATD. The G-MEDSS system also allows ACPs to enter medication recommendations and actions on the G-MEDSS reports to allow for the GP to comment on deprescribing recommendations. The G-MEDSS system can create a patient/carer summary report for patients or their carers at the time of the HMR. The CCDSS format of G-MEDSS allows for the patient information to be easily and accurately captured, recorded and translated to a standardised report format, which can be adapted to most patient settings. The specifics of the intervention in this study will involve ACPs producing G-MEDSS reports about their HMR patients to send together with the HMR report (i.e. as part of Step 3, Fig. 1) to the patient’s GP, and providing the patient/carer G-MEDSS reports to the patients or their carers. ACPs in the comparison group will conduct HMRs for their patients without using the G-MEDSS system (usual care).

#### Outcomes and follow-up

The primary outcome will be any reduction of anticholinergic and/or sedative medication use, as measured by DBI, between baseline and 3-months (binary outcome). Previous studies consistently observe an association between increasing DBI and impaired physical function [29]. The degree of exposure that results in a clinically significant change has been estimated to be a difference in DBI of approximately 0.5 [44]. Our previous study found that DBI is reduced in usual care HMR in 8.9% of patients by a median value of 0.28 [45], and an increase in this proportion by 10% was seen as a clinically meaningful effect on a population level. It was not feasible to power the study to primarily investigate impact on multifactorial measures of physical function.

Secondary outcomes will include:

- Recommendations to reduce anticholinergic and or sedative medications as measured by DBI in the HMR report and/or the G-MEDSS report;
- Prevalence of deprescribing (cessation and/or dose reduction) any medication
- Changes in clinical outcomes (physical function, cognitive function, falls, and institutionalisation)
- Changes in patient process outcomes (medication adherence and physician visits)
- Mortality at 3-months
- A subgroup analysis (of the above outcomes) for people living with and without dementia

All ACPs will be required to collect additional information of their HMR recipients at baseline (during HMR interview) and at a 3-month follow-up visit. The 3-month follow-up has been added exclusively to this study for the purposes of data collection as the HMR process (usual care) does not involve a follow-up visit. The following outcomes will be assessed of the patient participants enrolled in the study: medication adherence, cognitive impairment, functional status, falls, institutionalisation and the number of physician visits. Mortality data will be captured by the ACP participants at 3-month follow-up (Table 1).

**Table 1:** Patient participant data to be collected during the study

	Measure (s)	Baseline^a^	3-months
Demographics			
Age		✓	
Sex		✓	
Ethnicity		✓	
Marital Status		✓	
Education Status		✓	
Geographic remoteness/locality	PhARIA	✓	
Medication Profile			
Current medication list (prescribed, OTC and complementary, regular or PRN)		✓	
Changes to medication list (e.g. additions, cessations, ↑↓dose)			✓
Ceased medications		✓	✓
Adherence	MGL	✓	✓
Attitudes towards deprescribing^b^	rPATD, rPATDCog	✓	
Anticholinergic and sedative burden	DBI	✓	✓
Comorbidity and Physical Function			
Comorbidities	FCI	✓	✓
Cognition	Mini-Cog© [46]	✓	✓
Independent activities of daily living	NHCCSFSI	✓	✓
Physical Function	SPPB	✓	✓
Falls History		✓	✓
Others			
Institutionalisation (e.g. hospitalisation)		✓	✓
Physician visits (e.g. GP or specialist appointments)			✓
Mortality			✓
Goals of care^b^		✓	

^a^at the time of the HMR interview; ^b^only for patients in the intervention arm of the study

*PhARIA* The Pharmacy Access/Remoteness Index of Australia – quantifies degree of remoteness (geographic and professional) [47], *OTC* Over-the-counter, *PRN* when required, *MGL* Morisky, Green, Levine Scale [48], *rPATD* revised Patients’ Attitudes Towards Deprescribing [34], *rPATDCog* Revised Patients’ Attitudes Towards Deprescribing for people with Cognitive impairment [35], *DBI* Drug Burden Index [28], *FCI* Functional Comorbidities Index [49], *NHCCSFSI* National Home and Community Care Services Functional Screening Instrument [50], *SPPB* Short Physical Performance Battery [51], *GP* General practitioner

↑↓ = changes

The Morisky Green Levine Scale will be used to measure self-reported patient medication adherence [48]. Cognitive impairment will be assessed using the Mini-Cog© [46, 52–54]. The National Home and Community Care services Functional Screening Instrument (NHCCSFSI) (part one) and the Short Physical Performance Battery (SPPB) will be used to assess the functional status of patient participants [50, 51]. Details regarding scoring of these measures used in this study can be found in Additional file 1.

Data to calculate the Functional Comorbidity Index (FCI) will be collected from the patients. The FCI is a sum of 18 self-reported comorbid conditions with a score of 0–18. The FCI will also be used to measure comorbidities that predict physical function in older adults. A higher FCI score indicates greater morbidity and is associated with poorer physical function [49].

Falls (any fall in the last 12 months at baseline and number of falls at 3-months), institutionalisation (admission to hospital in the last 12 months at baseline and number of days admitted to hospital, nursing home or respite care at 3-months) and the number of physician visits (GP and specialist) will be captured over 3 months by providing patients and/or their carers a calendar to self-record events that will be collected by ACPs.

#### Sample size

The sample size calculation for this study is based on a feasibility study of implementing The Drug Burden Index Calculator© report into the HMR service [25]. In the feasibility study, 18 pharmacists recruited 100 patients (average cluster size 5.6) where 25 (25%) patients had a reduction in their DBI score, 7 (7%) patients had an increase in their DBI score, whilst the remaining 68 (68%) had no change in DBI score. The estimated intra-cluster correlation (ICC) was 0.07. With a sample size of 500 participants, we will have 80% power to detect a 10% difference between the intervention and comparison groups, corresponding to 20% of participants in the intervention group and 10% of participants in the comparison group having a reduction in DBI, assuming a 5% significance level (two-sided) and an Intra-cluster Correlation Coefficient (ICC) = 0.07. This corresponds to a relative reduction of 2, therefore we will be powered to detect a difference corresponding to twice the reduction in the intervention compared to the control.

As of March 2017, there were 2374 general registered ACPs practising in Australia. To reach the estimated clustered sample size of 500 patients, approximately 120 pharmacists will need to be recruited, 60 pharmacist participants in each of the intervention and comparison groups (allowing for 20% dropout rate – estimate based on the previous study [25]). Each ACP will be required to recruit 5–10 participants to achieve an average of 5 patients per ACP (total of 500 patients).

Using published national statistics, we estimate that 10–31% (*n* = 50–155) of recruited patients will have a diagnosis of dementia and subgroup analyses is planned on these participants [10].

#### Statistical analyses

The primary analyses will be conducted using an ‘intention-to-treat’ approach and will be reported according to the guidelines of the Consolidated Standards of Reporting Trials (CONSORT) 2010 statement. Descriptive statistics (means and proportions) will be used to report the demographics of the study populations (ACPs and patient participants) at baseline. Binary continuous and count outcomes will be analysed using logistic, linear and negative binomial regression models, respectively. All models will include a covariate for the intervention group, with a random effect for the clusters (ACPs). Analyses will provide an estimate of the difference between groups, 95% confidence intervals (CI) and *p*-values. All statistical tests will be two-tailed, and p-values of < 0.05 will be deemed statistically significant. Appropriate model checking will be conducted. The statistician performing the data analysis will be blinded to the identity of each treatment group.

Secondary analyses will include:

- Where appropriate, to conduct further adjusted analyses, covariates will be included for the patients’ baseline value (for that outcome) and any baseline characteristic for which there is evidence of imbalance between intervention and comparison groups;
- A subgroup analysis for people living with dementia;
- For clinical outcomes, assessments of associations between change in DBI or a deprescribing of a medication, using the same statistical methods as described above, but with the intervention group removed from the model and replaced by change in DBI or deprescribing of medication;
- Association between rPATD and deprescribing in overall patient population.

To analyse the responses to the rPATD questionnaire, factor scores will be created for each of the 4 factors (appropriateness, burden, concerns about stopping and involvement) as previously described [34]. Likert responses to the global question ‘I would be willing to stop one or more of my medications if my doctor said it was possible’ will be dichotomised into those that agree (strongly agree and agree) and those that are unsure/disagree (unsure, disagree, strongly disagree). Factor scores and responses to the global questions will be compared to deprescribing outcomes (DBI and prevalence of deprescribing) using Mann-Whitney and χ^2^ tests.

To analyse the free-text goals of care for patients (or carers) in the intervention arm (entered into G-MEDSS), the data will be transferred to NVivo qualitative data analysis software (QSR International Pty Ltd. Version 12, 2018) and analysed thematically to assess the goals of care that patients have for their medications and clinical conditions. All goals will be grouped into type of goal (health-related or medication-related) by two investigators, and differences will be discussed until consensus is reached.

### Stage II: process evaluation

**Fig. 4:**
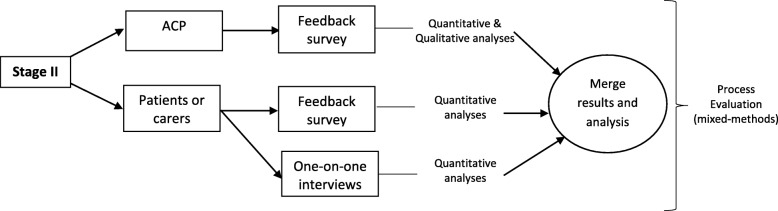
Stage II: Process evaluation of the intervention - flow of patients (or carers) and Accredited Clinical Pharmacists (ACP) through Stage II

The process evaluation will aim to evaluate the utility of G-MEDSS in the HMR service from the perspectives of ACPs and patients (or carers), and run alongside (in parallel to) Stage I. Specifically, the process evaluation will obtain the barriers and facilitators to the intervention to understand the factors that may impact on wider implementation (Fig. 4). The process evaluation will be guided by Moore et al.: a mixed-methods descriptive design where quantitative and qualitative data will be collected and triangulated to provide complementary insights of the ACPs and patients (or carers) [55]. Stage II will include both ACP and patient participants (or carers) who were randomised to the intervention arm of the study to evaluate the utility of G-MEDSS in the HMR process.

#### Recruitment of participants

ACPs who have recruited and completed follow-up of 5–10 patient participants (or carers) will be provided an opportunity to complete and return a feedback survey. The perspective and experiences of patients will be obtained using patient feedback surveys and one-on-one interviews. All patients (or carers) who have consented to participate in the study and have completed the baseline interview will be sent a feedback survey to complete and return.

#### Feedback survey

The ACP feedback survey consists of questions relating to the use of G-MEDSS during medication review and asks ACPs to comment on communicating patient goals of care, anticholinergic and sedative medication use and patient’s attitudes towards deprescribing in medication review reports to the GP. The feedback survey consists of open and closed-ended questions relating to the utility of G-MEDSS overall, is based on a previous study, consists of up to 18 questions, and ACPs will use a 4 or 5-point Likert scale, dichotomous responses and multiple-choice items (with options to provide more detail in free-text responses) to respond [31].

The feedback survey was developed for the patients (or carers) based on a study by Moon et al. using a literature search on existing surveys and interviews with pharmacists to identify relevant topics related to consumer satisfaction with pharmacist-led medication reviews [56]. These were addressing medication related-needs, pharmacist performance for engaging the consumer, and overall satisfaction. The survey consists of 10 questions that use a 4-point Likert scale to respond. Seven additional items designed by the research team will be included to assess patient (or carers) views towards G-MEDSS.

#### Qualitative interviews

Patients (or carers) will later be invited to participate in a semi-structured one-on-one interview (with MS via telephone). A semi-structured interview guide is the method chosen, as this was considered the most suitable method to obtain data from patients (or carers) with or without dementia [57]. The interview guide was based on a comprehensive theoretical paper on person-centred communication in consultations that is concordant with the individuals’ values, needs and preferences [58]. ACPs will not be interviewed as part of this process evaluation, as the data collected from the open-ended questions in the survey will be sufficient to ascertain the ACPs perspectives.

#### Mixed-methods analyses

In order to reduce bias, analyses of Stage II will commence before the analyses of Stage I. An integrated mixed-methods approach will allow for data triangulation and analyses. Data from the feedback surveys will be analysed using descriptive statistics. All patient interviews will be audio recorded and transcribed verbatim. NVivo qualitative data analysis software (QSR International Pty Ltd. Version 12, 2018) will be used for data management and each patient (or carer) participant will be assigned a number for anonymity. The qualitative data will be analysed using a descriptive approach via content analyses. Findings from the feedback surveys and interviews will be triangulated at the interpretation stage and will include discussions with the research team [59].

## Discussion

There is a current need to optimise deprescribing of inappropriate medications in older adults and it is important to involve collaboration with patients within this process [60]. CCDSS for medication management can be considered an effective method for reducing inappropriate prescribing and ADEs in older adults, however there is limited data on the real effect on patient outcomes [61]. Consolidating patient preferences and perspectives on outcomes and goals of therapy is important to guide decisions to reduce or stop medications [62]. There is limited evidence on the effect of goal-directed medication management on prescribing and clinical outcomes. The G-MEDSS is a novel CCDSS intervention that provides clinical decision support for healthcare practitioners, incorporating the patients’ goals and preferences during the medication review process. This study aims to evaluate the implementation of a CCDSS in HMRs to deprescribe medications, and to assess the effect of deprescribing on clinical outcomes.

### Strengths

This study has several strengths. The cluster-randomised trial design is known as the gold-standard study design for evaluating healthcare interventions. Additionally, we have integrated the intervention into usual HMR practice to increase the generalisability of the results, and to allow a more accurate estimation of the intervention’s effectiveness. Finally, the Stage II process evaluation of the study will allow explanation of any discrepancies between expected and observed outcomes, understanding of how context influences outcomes, and may provide insights to aid wider implementation into clinical practice [63].

### Limitations

There are several anticipated limitations within this study. Inherent biases in the sample population of ACPs and patients may affect the results of the study. ACPs usually work independently, therefore it is difficult to recruit ‘clinics’ of ACPs, similar to other cluster-randomised trials that involve general practice clinics. ACPs are also not blinded to the study group, therefore ACPs in the comparison arm may change their behaviour or clinical practice and this may not be reflective of true usual practice. On the other hand, ACPs in the intervention arm may recruit ‘clinically interesting’ patients that may ‘benefit’ from the intervention. To account for and to try to minimise these biases, all ACPs enrolled in the study will be required to undergo training (outlined above). The study is primarily powered to assess whether the intervention will reduce anticholinergic and sedative medication exposure, measured using DBI, after 3 months. It is not powered to assess differences in clinical outcomes, such as physical and cognitive function, even though this data will be captured. Therefore, we may not be able to detect a difference between the intervention and control groups regarding these clinical outcomes. The study will not collect quality of life measures for patients enrolled in the study or economic data on the impact of the intervention.

Regarding the process evaluation, the perspectives of the GPs will not be assessed. This may limit the interpretation of implementation of the intervention in the study and in clinical practice.

## Supplementary information

**Additional file 1.** Detailed methods on measurement of outcomes.

## Data Availability

Not applicable.

## Abbreviations

ACP: Accredited Clinical Pharmacist
ADE: Adverse Drug Events
CCDSS: Computerised Clinical Decision Support Systems
CI: Confidence Intervals
CONSORT: Consolidated Standards Of Reporting Trials
DBI: Drug Burden Index
FCI: Functional Comorbidity Index
G-MEDSS: Goal-directed Medication review Electronic Decision Support System
GP: General Practitioners
HMR: Home Medicines Review
ICC: Intra-cluster Correlation Coefficient
MCQ: Multiple Choice Questions
NHCCSFSI: National Home and Community Care services Functional Screening Instrument
RCT: Randomised Clinical Trial
rPATD: revised Patient Attitudes Towards Deprescribing
SPPB: Short Physical Performance Battery
