# Correction to: Implementation of the goal-directed medication review electronic decision support system (G-MEDSS)© into home medicines review: a protocol for a clusterrandomised clinical trial in older adults

**DOI:** 10.1186/s12877-020-01681-x

**Published:** 2020-10-02

**Authors:** Lisa Kouladjian O’Donnell, Mouna Sawan, Emily Reeve, Danijela Gnjidic, Timothy F. Chen, Patrick J. Kelly, J. Simon Bell, Sarah N. Hilmer

**Affiliations:** 1 NHMRC Cognitive Decline Partnership Centre, Northern Clinical School, Faculty of Medicine and Health, The University of Sydney, Sydney, NSW Australia; 2 Departments of Clinical Pharmacology and Aged Care, Kolling Institute of Medical Research, Royal North Shore Hospital, St Leonards, New South Wales 2065 Australia; 3 Geriatric Medicine Research, Faculty of Medicine, and College of Pharmacy, Dalhousie University and Nova Scotia Health Authority, Halifax, Canada; 4 College of Medicine, University of Saskatchewan, Saskatchewan, Canada; 5 Quality Use of Medicines and Pharmacy Research Centre, School of Pharmacy and Medical Sciences, Division of Health Sciences, University of South Australia, Adelaide, South Australia Australia; 6 Sydney Pharmacy School, Faculty of Medicine and Health, The University of Sydney, Sydney, NSW Australia; 7 Charles Perkins Centre, The University of Sydney, Sydney, NSW Australia; 8 School of Public Health, Faculty of Medicine and Health, The University of Sydney, Sydney, NSW Australia; 9 Centre for Medicine Use and Safety, Faculty of Pharmacy and Pharmaceutical Sciences, Monash University, Parkville, Victoria Australia; 10 School of Public Health and Preventive Medicine, Faculty of Medicine, Nursing and Health Sciences, Monash University, Melbourne, Victoria Australia; 11 Sansom Institute, School of Pharmacy and Medical Sciences, University of South Australia, Adelaide, South Australia Australia

**Correction to: BMC Geriatrics (2020) 20:51**

**https://doi.org/10.1186/s12877-020-1442-2**

Following publication of the original article [1], we have been notified that there is a mistake in author’s first and last names’ order.

Currently author’s first and last names are: Simon J. Bell.

It should, however, be: J. Simon Bell
