# Supplementary material for: Implementation of the Goal-directed Medication review Electronic Decision Support System (G-MEDSS)© into home medicines review: a protocol for a cluster-randomised clinical trial in older adults
Source: BMC Geriatr. 2020 Feb 12;20:51. doi: 10.1186/s12877-020-1442-2 (PMC7017507; doi:10.1186/s12877-020-1442-2)
Supplement: Supplementary file 1 — Additional file 1. Detailed methods on measurement of outcomes. [file 12877_2020_1442_MOESM1_ESM.docx]

**Appendix 2 – Detailed methods on measurement of outcomes**

Adherence:

The Morisky Green Levine Scale will be used to measure self-reported patient medication adherence.(1) This is a 4-item scale using dichotomous responses. The number of ‘Yes’ responses are counted: 0 ‘yes’ responses is considered ‘high adherence’, 1-2 ‘yes’ responses is considered ‘medium adherence’, and 3-4 ‘yes’ responses is considered ‘low adherence’.

Cognitive Impairment:

Cognitive impairment will be assessed using the Mini-Cog©.(2-4) The Mini-Cog is a brief, cognitive screening test used to evaluate cognition in older adults. It consists of a three-word recall task and the clock drawing test. The scoring involves assigning a score of 0 to 3 points on the word recall task, for the correct recall of 0, 1, 2 or 3 words, respectively. For consistency amongst the ACPs, Word List Version 1 (‘Banana’, ‘Sunrise’, ‘Chair’) will be used for the word recall task for all patient participants. The clock drawing test is scored as being either ‘normal’ or ‘abnormal’. To interpret the Mini-Cog, the 3-item recall and clock drawing scores are added together. A positive test on the Mini-Cog (i.e. patient is screened as having cognitive impairment or dementia) is considered if the total score is 3, 4, or 5. (5)

Physical Function:

The National Home and Community Care services Functional Screening Instrument (NHCCSFSI) (part one) and the Short Physical Performance Battery (SPPB) will be used to assess the functional status of patient participants.(6, 7) The NHCCSFSI is designed to measure and quantify key areas in which a person requires assistance with daily living. Part one contains seven items that are asked of the patient or carer that screen for self-care and domestic functioning. Each item is given a score of 0, 1 or 2, where 0 is ‘unable to complete the task’, and 2 is ‘able to complete the task without help’. Part two contains two items that are asked of the ‘screener’ to judge based on interviewing the patient, which include items on memory and behaviour (subjective assessment). For the purposes of this study, only part one (self-care and domestic functioning – objective measures) will be used to avoid perceived subjective bias (arising from the clustered-RCT design) from part two. To assess whether the patient requires assistance with activities of daily living, the number of items that have scored 2 will be counted. If a patient can do less than 3 activities without assistance (i.e. they scored 2 for 2 items or less), they are considered to require assistance in activities of daily living. The SPPB will also be used to assess functional status and physical performance. This composite measure incorporates three tasks: balance tests, gait speed tests and chair stand tests. All ACPs will be provided the equipment (e.g. 4-metre measure) and training to conduct these tests. All tests are timed and scored accordingly, with each task scored out of 4 and the scores from the three tests summed to give a total (minimum score = 0 and maximum score = 12). A higher score indicates a higher level of function and a lower score indicates a lower level of function. A change of 1 point on the SPPB is considered to be a substantially meaningful change.(8)

References:

1. Morisky DE, Green LW, Levine DM. Concurrent and predictive validity of a self-reported measure of medication adherence. Med Care. 1986;24(1):67-74.

2. Borson S, Scanlan J, Brush M, Vitaliano P, Dokmak A. The mini-cog: a cognitive 'vital signs' measure for dementia screening in multi-lingual elderly. Int J Geriatr Psychiatry. 2000;15(11):1021-7.

3. Setter SM, Neumiller JJ, Johnson M, Borson S, Scanlan JM. The Mini-Cog: a rapid dementia screening tool suitable for pharmacists' use. Consult Pharm. 2007;22(10):855-61.

4. Borson S, Scanlan JM, Chen P, Ganguli M. The Mini-Cog as a screen for dementia: validation in a population-based sample. Journal of the American Geriatrics Society. 2003;51(10):1451-4.

5. Borson S. Mini-Cog(c) Screening for Cognitive Impairment in Older Adults 2019 [Available from: <https://mini-cog.com/>.

6. Green J, Eagar K, Owen A, Gordon R, Quinsey K. Towards a Measure of Function for Home and Community Care Services in Australia: Part 2 - Evaluation of the Screening Tool and Assessment Instruments. Australian Journal of Primary Health. 2006;12(1):82-90.

7. Guralnik JM, Simonsick EM, Ferrucci L, Glynn RJ, Berkman LF, Blazer DG, et al. A short physical performance battery assessing lower extremity function: association with self-reported disability and prediction of mortality and nursing home admission. J Gerontol. 1994;49(2):M85-94.

8. Treacy D, Hassett L. The Short Physical Performance Battery. J Physiother. 2018;64(1):61.
